# A Japanese version of the Pearlin and Schooler’s Sense of Mastery Scale

**DOI:** 10.1186/s40064-015-1186-1

**Published:** 2015-08-07

**Authors:** Taisuke Togari, Yuki Yonekura

**Affiliations:** Faculty of Liberal Arts, The Open University of Japan, Chiba, Japan; Department of Hygiene and Preventive Medicine, Iwate Medical University, Morioka, Japan

**Keywords:** Sense of mastery, Reliability and validity, Scale development, Japanese

## Abstract

The aims of this research were to develop a Japanese version of Pearlin and Schooler’s Sense of Mastery Scale (SOMS) and evaluate its reliability and validity. This survey targeted 4,000 men and women aged 25–74 living in Japan as of January 1, 2014, categorized them according to the region and size of the city in which they lived, randomly extracted 200 municipalities, and randomly extracted individuals after categorizing for sex and age based on the resident registries of each municipality. 2,067 survey responses were collected (response rate 51.7%). We used weighted 7-item (SOMS-7) and 5-item (SOMS-5) versions that excludes two reverse items (item6 and 7) from SOMS-7 of the SOMS. From the item analysis, the item-total correlation coefficients of the two reverse items (items 6 and 7) were .03 and .34. The Cronbach’s alpha coefficient was also .69 in SOM-7 and .77 in SOMS-5. The partial correlation coefficients between SOMS and the sense of coherence, mental health inventory, self-rated health, and life satisfaction were all significant (p < 0.001). The SOMS showed high construct validity. SOMS-5 has sufficient reliability.

## Background

The sense of mastery, which is conceptualized as a coping mechanism to reduce stress, was proposed by Pearlin and Schooler (Pearlin and Schooler 1978). According to Pearlin, mastery is a self-belief, a conviction that people are able to control the important circumstances currently impinging on their life (Pearlin 2010). That sense of mastery is a protective resource with a stress-moderating capacity that has been repeatedly tested since the Sense of Mastery Scale (SOMS) was developed by Pearlin et al. in the 1970s (Pearlin and Bierman 2013). The sense of mastery also tends to develop from a background of successful attainment of socially prized goals, and therefore is related to advantageous socio-economic status (Pearlin et al. 2007).

In Japan, self-control-concepts related to one’s health have been proposed, with the most well-known being the locus of control (Wallston et al. 1976). However, the sense of mastery is little known in Japan. Locus of control has more limited focus on the control of conditions that individuals regard as important determinants of their own personal lives (Pearlin and Pioli 2003). Conversely, the sense of mastery focuses on all environmental conditions (Pearlin and Pioli 2003).

Despite much research on the use of the SOMS in Europe and the United States, a Japanese version has not yet been developed. Therefore, the aim of this research was to develop a Japanese version of Pearlin’s SOMS, and examine its reliability and validity.

## Methods

### Forward translation and back translation

In creating the Japanese version of the SOMS, we first obtained the necessary permission from Prof. Pearlin. Second, our colleagues conducted a forward translation. Third, a bilingual person performed a back translation. Finally, Pearlin checked the back-translated items, and the items were revised based on his comments.

### Sample

This survey targeted 4,000 men and women aged 25–74 living in Japan as of January 1, 2014, categorized them according to the region and size of the city in which they lived, randomly extracted 200 municipalities, and randomly extracted individuals after categorizing for sex and age based on the resident registries of each municipality. This method was thereby a stratified two-stage extraction method. During the period from February to March 2014, in which self-administered questionnaires were sent out, 2,067 responses were collected using a leaving method (response rate 51.7%). Cases with inadequate answers about the SOMS were extracted. The final sample size was 954 male and 1,101 female respondents, with a mean age (SD) of 50.0 (14.2). This research was approved by the institutional review board of The Open University of Japan.

### Variables

#### Sense of mastery scale

The SOMS version used was Pearlin and Schooler’s 7-item version. This version included statements such as “There is really no way I can solve some of the problems I have” and “Sometimes I feel that I’m pushed around in life”, and used a 4-point Likert scale with possible responses of “strongly disagree”, “somewhat disagree”, “somewhat agree”, and “strongly agree” (Pearlin and Schooler 1978).

#### Sense of coherence

The sense of coherence (SOC) refers to the sense of looking at or confronting one’s life and environments with the ability to cope with stressors. A 13-item SOC Scale (SOC-13) was used. The reliability and validity of this scale has been demonstrated (Eriksson and Lindström 2005). The total score was tallied using a 7-point semantic differential to serve as the SOC score. Cronbach’s alpha coefficient = .84

#### Mental health inventory

Mental health was measured using the Japanese version of the five-item Mental Health Inventory (MHI5) related to depression and anxiety from the Medical Outcome Study Short Form (Rumpf et al. 2001). The reliability and criterion-related validity of this scale were verified for the Japanese population aged 16 or older (Yamazaki et al. 2005).

#### Self-rated health

Self-rated health (SRH) was measured on a 5-point scale ranging from “poor” to “excellent” in response to questions such as, “How is your present health?” SRH has been shown to affect survival rates controlled for objective health status (Idler and Benyamini 1997).

#### Life satisfaction

Life satisfaction was measured with a 5-point scale with the responses, “unsatisfactory”, “rather unsatisfactory”, “neither unsatisfactory nor satisfactory”, “rather satisfactory”, and “satisfactory” in response to questions such as, “How is the entirety of your life?”

### Statistical methods

Two versions of the SOMS have been used in previous research, a 7-item version (SOMS-7) and a 5-item version (SOMS-5) that excludes two reverse items from SOMS-7. In recent years, SOMS-5 has been used frequently because of its good reliability (Deeg and Huisman 2010). In this study, both versions were weighed. First, reliability analyses [Cronbach’s Alpha coefficients (Alpha)] and item-total correlations were computed. Second, confirmatory factor analysis by structural equation modeling was performed to examine factor validity. Third, sex and age group differences were examined using Student’s *t* test and one-way ANOVA. Finally, partial correlation analysis, controlled for age and sex, was performed to examine the relationships between the SOMS and related factors including SOC, MHI5, SRH and life satisfaction, as the construct validity. All analyses were conducted using IBM SPSS Statistics 22.0.

## Results

From the item analysis (Table 1), the item-total correlation coefficients of the two reverse items (items 6 and 7) were .03 and .34. The Cronbach’s alpha coefficient was also .69 in SOM-7 and .77 in SOMS-5. Confirmatory factor analysis regarding one-factor model resulted in follow model fit indices; CFI = .86 and RMSEA = .115 for SOMS-7, CFI = .98 and RMSEA = .074 for SOMS-5. Mean values (SD) were 19.1 (3.3) for SOMS-7 and 13.8 (2.9) for SOMS-5. There were no differences between sexes in either scale. However, a one-way ANOVA showed significant differences (F = 3.63, p < 0.001 in SOMS-7; F = 7.28, p < 0.001 in SOMS-5). Results of multiple comparison tests are shown in the Table 1. Partial correlation coefficients between the SOMS and related variables are shown in the Table 1. All coefficients were significant (p < 0.001).

**Table 1:**
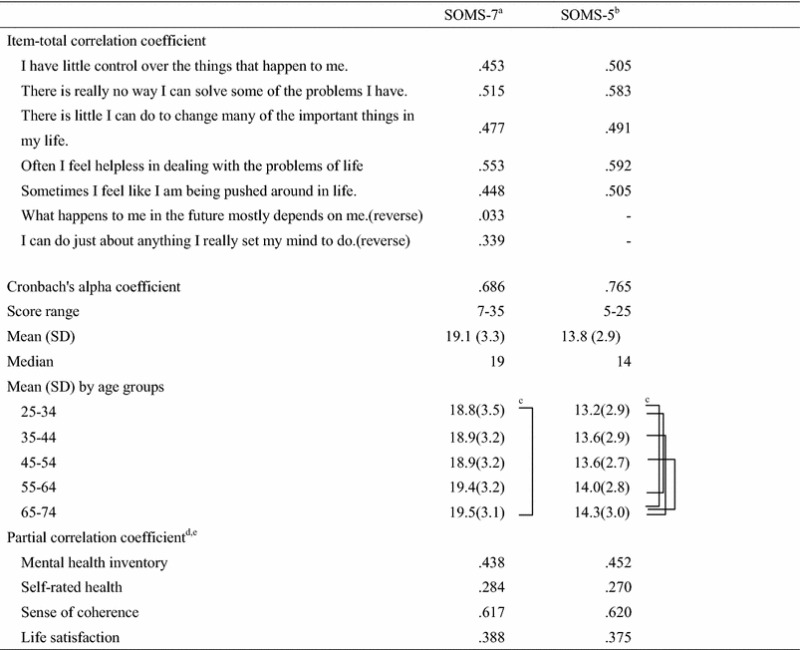
**Reliability and validity of sense of mastery scale Japanese version**

^a^7-item version of sense of mastery scale.

^b^5-item version of sense of mastery scale.

^c^Tukey's multiple comparison test (p < .05).

^d^Controlled for gender and age.

^e^All correlation coefficients were significant (p < .001).

## Discussion

In this research, the SOMS had high construct validity; namely, the correlation coefficients between the SOMS and SOC were high, indicating a certain level of concurrent validity. SOC as well as the sense of mastery had stress-moderating effects (Idler and Benyamini 1997). Moreover, SOMS-5 and SOMS-7 were associated with MHI-5, SRH, and life satisfaction as mental health outcomes. Because the sense of mastery is a mediator of stress (Deeg and Huisman 2010), these results indicate that the SOMS has construct validity.

However, the Cronbach’s alpha coefficient for SOMS-7 had a moderately low value. In addition, model-fit indicators for the confirmatory factor analysis were moderately low. Therefore, we need to treat findings regarding SOMS-7 with caution.

SOMS-5 does have sufficient reliability and factor validity. Because of its frequent usage in recent years, its Japanese version can be used without difficulty.

This study had some limitations. This study design was cross-sectional. In future research, we should conduct follow-up surveys and study predictive validity and test–retest reliability. As directions for future study, it is also important to study reproducibility, the predictability of health-related indicators from the perspective of construct validity, and the stress-buffering effects by way of longitudinal research.
